# Compliance Trends in a 14-Week Ecological Momentary Assessment Study of Undergraduate Alcohol Drinkers

**DOI:** 10.1177/10731911231159937

**Published:** 2023-03-13

**Authors:** Andrea L. Howard, Megan Lamb

**Affiliations:** 1 Carleton University, Ottawa, Ontario, Canada

**Keywords:** ecological momentary assessment, compliance, longitudinal, university students

## Abstract

High compliance is a priority for successful ecological momentary assessment (EMA) research, but meta-analyses of between-study differences show that reasons for missed prompts remain unclear. We examined compliance data from a 14-week, 182-survey EMA study of undergraduate alcohol use to test differences over time and across survey types between participants with better and worse compliance rates, and to evaluate the impact of incentives on ongoing participation. Participants were *N* = 196 students (65.8% female; *M*_age_ = 20.6). Overall compliance was 76.5%, declining gradually from 88.9% to 70% over 14 weeks. Declines were faster in participants with lower overall compliance, but we found no demographic, personality, mental health, or substance use differences between participants with better versus worse compliance rates. Compliance varied by survey type, and unannounced bonus incentives did not impact compliance rates. Participants completed fewer surveys the week after winning a gift card. We offer recommendations for designing future EMA studies.

Ecological momentary assessment (EMA), also known as experience sampling or ambulatory assessment, is an approach to study design that collects real-time data on participant behaviors, emotions, and experiences in their natural environments (Shiffman et al., 2008) and in ways that intrude minimally on participants’ daily lives (Burke et al., 2017). At their inception in the 1980s, participants carried pagers and completed paper surveys whenever they were “beeped” by the study team (Csikszentmihalyi & Larson, 1987). Technological advances in recent years mean that EMA studies can take place on participants’ personal smartphones with great ease and little expense. EMA has unsurprisingly grown in popularity, and the share of published scientific articles using these methods was over 6 times higher in 2021 compared with 2008, when Shiffman and colleagues published their summative annual review on EMA.^1^

Compared with in-lab experimental methods and even to longer-term diary and longitudinal studies, the short-term windows of data collection throughout the day result in many missed assessments that threaten the validity of inferences drawn from EMA studies (Sun et al., 2020). Understanding *compliance* with EMA protocols has generated considerable research interest and at least four meta-analyses of study and participant characteristics (Jones et al., 2019; Ottenstein & Werner, 2022; Williams et al., 2021; Wrzus & Neubauer, 2022). However, between-study comparisons have shown no consistent differences in compliance across studies as a function of design factors (e.g., prompt frequency, length of study). Potential factors driving compliance differences may be filtered out during the recruitment phase, suggesting the need for within-study tests of differences in compliance. In the present exploratory and descriptive study, we take advantage of data gathered from undergraduate drinkers over 14 consecutive weekends (Thursday to Sunday) to evaluate differences over time between participants with better and worse compliance rates, and to evaluate the potential impact of different types of incentives on ongoing participation.

## Between-Study Similarities in Compliance

Meta-analyses show that on average, participants in EMA studies respond to 75% to 82% of the prompts they receive over the course of their study involvement (Jones et al., 2019; Ottenstein & Werner, 2022; Williams et al., 2021; Wrzus & Neubauer, 2022). Studies with fewer prompts per day and shorter surveys generally did not have better compliance rates than studies with more prompts per day and longer surveys, consistent with experimental evidence (Eisele et al., 2022; Hasselhorn et al., 2022). Other design factors showing no effect on compliance included type of device (e.g., personal smartphone vs. study-loaned device), whether prompts were at fixed versus random times of day, and whether studies included event-contingent responding. Paid samples had slightly better compliance than unpaid, but different strategies for payments—such as bonuses for completing more surveys—were not related to better compliance rates. Gender, age, and clinical versus community sample participant sources were also unrelated to compliance.

For researchers designing EMA studies, it is tempting to conclude that a high compliance rate is achievable irrespective of design factors in the study. However, studies reviewed across meta-analyses had compliance rates ranging as low as 9.8% up to 100%. Compliance rates below 80%—a heuristic benchmark minimum for EMA studies (Jones et al., 2019)—were reported in nearly half of the studies reviewed by Wrzus and Neubauer (2022). A conclusion from their study was that self-selection is a key reason for the lack of between-study differences observed. Reasons that some people might not adhere to a study protocol likely discourage them from agreeing to participate in the first place. Tests of *within-study* differences are needed to establish whether in a single sample, there are any characteristics of participants or study design (e.g., length, type of survey, incentive structure) that might influence compliance rates.

Clues pointing to potential sources of differences in compliance and attrition over time are scarce. One EMA study explored this question intensively by equipping student participants with passive audio listening devices and coding their behavior and context in proximity to when EMA survey responses were requested (Sun et al., 2020). Simply being a participant who completed many versus few surveys over the course of the study allowed for nearly all completed reports to be correctly classified as complete. However, just 21% of *missing* reports were correctly classified as missing. Adding personality, social, and emotional variables coded from listening data did not improve correct classification of missing reports. Similarly, a recent study of young adults found only weak evidence that traits and behaviors such as aggression, delinquency, and alcohol use correlate with poorer compliance, and demographic measures were unrelated to compliance (Murray et al., 2022). Drawing on these observations, the first aim of our exploratory study was to describe and test for differences in available measures of demographic characteristics, personality, and mental health across groups of participants classified as more or less compliant based on their overall level of study engagement.

## Compliance in Longer Duration Studies

A surprising null finding from meta-analyses of EMA compliance is that longer duration studies do not have consistently poorer compliance than shorter studies. Two meta-analyses classified study durations as <1 week, 1 to 2 weeks, and 2 or more weeks in length and found no mean differences in compliance rates (Jones et al., 2019; Williams et al., 2021). One meta-analysis found a small negative correlation between compliance rate and the total number of study days (Ottenstein & Werner, 2022). The largest meta-analysis included just seven studies (out of several hundred) that reported total study lengths exceeding 30 days, and compliance rate was unrelated to study length (Wrzus & Neubauer, 2022). These results are difficult to reconcile with the universally acknowledged problems of attrition and missing data in longitudinal survey research and underscore the limits of a between-study approach to understanding EMA compliance. In an integrative data analysis of 10 EMA studies lasting 4 to 6 days, an overall compliance of 78% was close to the average rate reported in each meta-analysis, but compliance declined from a high of 83% on the first day to 73% on the fifth day (Rintala et al., 2019). Overall rates mask patterns of attrition and offer no insights suggesting who drops out, when and why they drop out, and how we might best encourage retention, especially for EMA studies lasting weeks or months.

The longer duration design of the present study offers a unique opportunity to explore compliance trends. We issued surveys on 56 days spread out over 14 weekends. At 13 surveys per weekend, participants had the opportunity to contribute as many as 182 responses. The longer the study, however, the more opportunities for attrition. In one 7-day EMA study of students participating in exchange for course credit, compliance above 80% on Day 1 dropped to around 65% on Day 4 (Silvia et al., 2013). A paid sample of first-year undergraduates had even worse compliance, with 70% missing 3 or more days’ worth of surveys over the course of a 7-day protocol (Bedard et al., 2017). As shown in meta-analyses, there were no reliable differences in compliance between studies using different designs, but these tests do not rule out within-study differences in compliance *trends* as a function of design characteristics. Analyses of trends can determine whether compliance wanes over time at the same or different rates, helping to identify people and design characteristics exhibiting the slowest rates of decline over time. Our study included surveys issued at 4 times per day, with variable response windows (2 and 4 hours), and one survey featuring a repetitive cognitive task. Accordingly, the second aim of our exploratory study was to describe and model rates of change in week-to-week compliance, within-week compliance, and need for reminders across respondent category and survey types each weekend.

## Financial Incentives

The sole criterion that predicted higher compliance in between-study meta-analyses was the use of incentives. Studies offering monetary incentives averaged compliance rates of 82.2%, significantly higher than studies offering nonmonetary incentives (77.5%) or no incentives (76.2%; Wrzus & Neubauer, 2022). However, considerable variability remains and half the studies in Wrzus and Neubauer’s meta-analysis with no incentives achieved a compliance rate above 80%, although none of these studies exceeded 28 days and most lasted for 1 week or less. In a three-sample study with long tracking periods ranging from 35 to 62 days, compliance rates were low when student participants received only course credit (32.8%) and higher when they received money (76.7%) or a prize (71.4%; Harari et al., 2017). Compliance rates also waned more rapidly in the credit-only versus compensation samples. Participants in the present study were all compensated at a rate of $1 per completed survey, with payments issued as gift cards on a weekly basis. However, our design also included two additional forms of compensation that allow us to test for potential effects of incentives on compliance: The first was the use of a “surprise” bonus payment: After about 5 and 10 weeks of participation, participants received unannounced $5 gift cards thanking them for their ongoing commitment to our study. The second was the use of daily prize draws: On Fridays, Saturdays, and Sundays, we randomly selected a $25 gift card winner from among the students who participated the previous day. Taking advantage of these supplemental forms of compensation, the third aim of our exploratory study was to test for differences in compliance rates before and after receiving prizes and bonuses to determine whether these strategies boosted compliance.

## The Current Study

We use data drawn from a 14-week ecological momentary assessment study of undergraduate alcohol drinkers to accomplish three aims: First, we classify participants into groups based on their overall study compliance rates and compare them on several demographic, personality, mental health, and substance use measures. Second, we visualize trends over time in compliance and model rates of change in week-to-week compliance, within-week compliance, and need for reminders across respondent categories and survey types. Third, we test whether compliance rates differ on weeks before and after participants received surprise bonuses and prizes. Our design is exploratory and no part of this study was preregistered. Sample size was restricted to the number available from our existing data. All data decisions and measures in the study are reported below. Supplemental materials, raw data, and analysis code are available on the Open Science Framework (OSF) page for this project (https://osf.io/d4ea8).

## Method

### Participants and Procedure

Participants were *n* = 196 undergraduate students attending a Canadian university in the Fall of 2021, recruited online and in-person from August 23 to September 17, 2021 (in-person classes had not fully resumed at this time). We hosted 10 Instagram “giveaways” of $100 gift cards every 2 to 3 days during recruitment. Between giveaway posts, we circulated recruitment notices, some of which were promoted posts (ads) that targeted Instagram users aged 18 to 25, located within 50 km of our university, and who listed the university among their tagged interests on Instagram. We also posted study information on Reddit, Facebook, and Twitter; placed paper postcards in high-traffic areas around campus (e.g., at welcome desks; on benches near student residences; on seats in classrooms); and participated in a virtual orientation week event for student clubs and activity groups.

Interested students were directed to complete an eligibility survey. Eligibility criteria included (a) being enrolled as an undergraduate student at our university, (b) aged 25 or younger, and (c) planning to drink at least twice a month during the Fall semester. Ineligible students who completed our screening survey were thanked for their time. Eligible students were invited to provide their name and university-assigned email address. A *reCAPTCHA* survey question was included at this stage to limit “bot” activity on our screening survey, and respondents who provided a nonuniversity email address were removed. Of the 1,021 responses to our screening survey, *n* = 342 were eligible to participate and received an invitation and personalized link to complete the intake survey. Of these, *n* = 301 participants provided informed consent and initiated an intake survey. We excluded 31 respondents at intake because they subsequently reported drinking or planning to drink less than 2 times per month, and another four were excluded due to incomplete demographic information.

Participants who initiated the intake survey received a $10 *Amazon.ca* gift code (hereafter referred to as “amazon cash”). We scrutinized intake responses for data quality issues using open-ended questions and total completion time. Criteria were that students provided sensible responses to open-ended questions, completed the survey in full and took at least 5 minutes to do so, and expressed an interest in participating in a follow-up EMA study. We invited *n* = 239 students to register by completing a secondary consent for the EMA study phase and providing a cell phone number to receive survey prompts (*n =* 204). Over a 5-week period, we enrolled *n =* 196 students (Supplement 1, Table S1 shows detailed week-by-week participant enrollment, compliance, and dropout numbers).

The EMA study phase consisted of 13 surveys per week for 14 weeks. Participants received text message alerts at the same times each day and had 2 to 4 hours to complete each survey. The first alert arrived in the *morning* (Thursday through Sunday; alert at 10:00 a.m.; available until 2:00 p.m.) and the next at *midday* (Thursday through Saturday; alert at 4:00 p.m.; available until 6:00 p.m.), followed by *early evening* (Thursday through Saturday; alert at 7:00 p.m.; available until 9:00 p.m.) and *late evening* surveys (Thursday through Saturday; alert at 11:00 p.m.; available until 3:00 a.m. the following morning). We also gave participants the option of completing a “going to sleep” survey each evening if they were planning to be asleep before the late evening survey launched at 11:00 p.m. Participants who entered their study ID number on our nightly going to sleep survey were treated as having responded to the late evening survey. Depending on branching options selected, morning surveys included up to 45 questions, and both evening surveys included 13 questions. The midday survey included a brief nine-item survey and a Stop Signal task with five trials and a median completion time of 4.25 minutes.

Students were compensated $1 in amazon cash for each survey they completed, with payments sent out every Tuesday. To boost retention and promote student engagement, we conducted daily draws awarding $25 in amazon cash to one winner per day, Friday through Sunday. Participants who completed at least one survey on the previous day (i.e., Thursday through Saturday) were entered into the draw. After Weeks 5 and 10, we also distributed two unannounced $5 bonuses to students who remained enrolled in the study.

### Measures

#### Demographic Characteristics

On our intake survey, students provided their age, gender identity, racial/ethnic background, parents’ education and income, frequency of cannabis and alcohol use in the past year, and indicators of food insecurity (details available in a data dictionary included on our OSF project page; https://osf.io/d4ea8).

#### Personality, Mental Health, and Substance Use

We include a detailed data dictionary of measures of personality, mental health, and substance use on our OSF project page. Briefly, we calculated average scores for depression (Center for Epidemiological Studies—Depression; CES-D; Andresen et al., 1994), anxiety (Generalized Anxiety Disorder; GAD; Spitzer et al., 2006), and life satisfaction (Satisfaction with Life Scale; Diener et al., 1985). Subscale scores were created for personality (i.e., extraversion, agreeableness, conscientiousness, negative emotionality, and open-mindedness; BFI-2-S; Soto & John, 2017) and impulsivity measures (i.e., negative urgency, perseverance, premeditation, sensation seeking, and positive urgency; Whiteside & Lynam, 2001). Participants also reported on their past-year frequencies of alcohol and cannabis use.

#### Weekly Overall Compliance

For each person, we summed the number of surveys they completed in a given week (i.e., 0–13), divided by the number of possible surveys (i.e., 13), and multiplied by 100 to arrive at a *weekly compliance rate*. Each week, we summed the weekly compliance rates across all participants and divided by the number of enrolled participants that week. The denominator varied with rolling enrollment and dropout each week.

#### Weekly Compliance by Survey Type

We separately calculated compliance rates for each person on each of the morning, midday, early, and late evening surveys. Participants could complete up to four morning surveys and three midday, early, and late evening surveys per week. For each person, we summed the number of surveys completed that week for a given survey type (i.e., 0 to 3 or 4), divided by the number of possible surveys (3 or 4), and multiplied by 100 to arrive at *survey-specific compliance rates*. For each survey each week, we summed the corresponding survey-specific compliance rates across all participants and divided by the number of enrolled participants each week. As with overall compliance, the denominator varied with rolling enrollment and dropout each week.

#### Reminders

We sent up to two text message reminders for each survey to encourage completion, delivered at the same time each day for each survey type and only to participants who had not yet completed their survey. For a given survey, anyone who started a survey after the first or second reminder was sent was recorded as needing one or two reminders, respectively, before completing the survey. We calculated mean numbers of reminders (overall and by survey) each week to examine trends over time in the need for reminders.

#### Responder Category

Because of our interest in using compliance rates to predict compliance trends—effectively “double-counting” our outcome variable—we devised a categorical measure of participants’ overall levels of compliance across the duration of our study. Cut points were selected based on heuristic benchmarks for acceptable levels of compliance balanced against the need to have comparable numbers of people in each category. We designated participants as *super* responders if they responded to more than 90% of our surveys; *good* if they responded to 75% to 89% of surveys; *adequate* if they responded to 50% to 74% of our surveys; and *poor* if they responded to fewer than 50% of our surveys.

#### Surprise Bonuses

We distributed surprise (unannounced) $5 gift card bonuses twice over the course of the study. These were not mentioned in the consent form and were not disclosed in advance to participants. Surprise bonuses were issued in Weeks 5 and 10 (for participants who began the study in Weeks 1 to 3) or Weeks 6 and 11 (for participants who began the study in Weeks 4 and 5). We recorded each participant’s weekly compliance for the weeks immediately preceding and following each bonus.

#### Daily Draws

Participants who completed at least one survey on a given Thursday, Friday, or Saturday were entered into a daily draw for a $25 gift card the next day. Over the course of the study, we distributed 42 gift cards to 38 people (four people won a gift card more than once). For each draw winner, we recorded their compliance rate from the week preceding their win, the week of their win, and the week following their win.

### Analysis Strategy

Descriptive and inferential analyses for this study were performed using R software (R Core Team, 2021) and packages *dplyr* (Wickham et al., 2022), *lme4* (Bates et al., 2015), *lmerTest* (Kuznetsova et al., 2017), *emmeans* (Lenth, 2022), and *jmv* (Selker et al., 2021). Visualizations were created with *ggplot2* (Wickham, 2016). We performed chi-square tests and one-way analyses of variance (ANOVAs) to examine demographic differences between responder types (super, good, adequate, poor) on categorical and continuous demographic, personality, and mental health measures. We used mixed effects regression models to estimate trends over time in compliance rates across weeks (1 to 14), survey types (morning, midday, early and late evening), and by responder type, permitting interactions between each of these measures. We probed significant interactions with simple slopes and contrasts. We conducted a 2 (within) × 4 (between) random-effects ANOVA to test for mean differences in weekly compliance pre-and post-bonus compensation by survey type. Finally, we performed one-way random-effects analyses of covariance (ANCOVAs) to test the impact of winning a daily draw on compliance rates before, during, and after the week of the draw win, controlling for study week.

## Results

### Compliance Summary

The final sample size for the EMA study was *n* = 196. This varied over recruitment weeks (1–5) where participants were enrolling and dropping out concurrently. Over the 14 weeks, we administered 30,940 surveys, and 23,661 were completed, giving an overall compliance rate of 76.5%. Based on each participant’s start date and number of surveys available to them (Week 1: 182 to Week 5: 130), we classified *n* = 29 as *poor*, *n* = 49 as *adequate*, *n* = 62 as *good*, and *n* = 56 as *super* respondents.

### Demographic Characteristics

Participants identified as female (65.8%; *n* = 129), male (29.6%; *n* = 58), or neither male nor female (4.6%; *n* = 9). In addition, 15 participants (7.7%) further identified as nonbinary, transgender, genderqueer, or agender. The sample was ethnically diverse: 6% identified as Black (*n* = 12), 2.5% as Indigenous (*n* = 5), 1.5% as Latinx (*n* = 3), 9.7% as multiple ethnicities (*n =* 19), 9.7% as South Asian (*n =* 19), 7.1% as Southeast Asian (*n =* 14), 2.5% as West Asian (*n* = 5), 0.5% selected an identity other than those listed (*n* = 1), and 60.2% identified as White (*n* = 118). Average combined parent income was 6.83, or an estimated $120,750 per year,^2^ slightly below the median two-parent household income in Ontario (inflation-adjusted; Munger et al., 2016).

On average, participants were 20.61 (*SD* = 1.50; range = 17–25) years old. We classified *n* = 22 participants (11.2%) as *food insecure* if they reported that they worried about or actually ran out of food and were unable to afford to eat balanced meals. Table 1 shows detailed demographic information for each responder group. None of the tests of demographic characteristics showed significant differences between responder types.

**Table 1.** Sample Characteristics by Respondent Type.

	Total	Responder category				χ^2^
Variable		Poor	Adequate	Good	Super	
	196	29	49	62	56	
*N*	*n* (%)	*n* (% within responder category)				
Gender						
Female	129 (65.8%)	21 (72.4%)	32 (65.3%)	42 (67.7%)	34 (60.7%)	χ^2^ = .31(3)
Male	58 (29.6%)	8 (27.6%)	16 (32.7%)	18 (29.0%)	16 (28.6%)	*p* = .957^a^
Other identity	9 (4.6%)	0 (0%)	1 (2%)	2 (3.2%)	6 (10.7%)	
College-educated parent(s)						
0 (No)	65 (33.2)	13 (44.8%)	16 (32.7%)	18 (29.0%)	18 (32.1%)	χ^2^ = 2.29(3)
1 (Yes)	131 (66.8%)	16 (55.2%)	33 (67.3%)	44 (71.0%)	38 (67.9%)	*p* = .515
Ethnicity						
Black	12 (6.1%)	4 (13.8%)	1 (2.0%)	4 (6.5%)	3 (5.4%)	χ^2^ = 4.28(3)
Indigenous	5 (2.6%)	0 (0%)	1 (2.0%)	2 (3.2%)	2 (3.6%)	*p* = .233^b^
Latinx	3 (1.5%)	0 (0%)	1 (2.0%)	1 (1.6%)	1 (1.8%)	
Mixed	19 (9.7%)	2 (6.9%)	7 (14.3%)	4 (6.5%)	6 (10.7%)	
South Asian	19 (9.7%)	1 (3.5%)	3 (6.1%)	11 (17.7%)	4 (7.1%)	
Southeast Asian	14 (7.1%)	2 (6.9%)	3 (6.1%)	5 (8.1%)	4 (7.1%)	
West Asian	5 (2.6%)	0 (0%)	1 (2.0%)	3 (4.8%)	1 (1.8%)	
White	118 (60.2%)	20 (69.0%)	32 (65.3%)	31 (48.3%)	35 (62.5%)	
Other identity	1 (0.5%)	0 (0%)	0 (0%)	1 (1.6%)	0 (0%)	
Food insecure						
0 (secure)	174 (88.8%)	26 (89.7%)	42 (85.7%)	54 (87.1%)	52 (92.9%)	χ^2^ = 1.59(3)
1 (insecure)	22 (11.2%)	3 (10.3%)	7 (14.3%)	8 (12.9%)	4 (7.1%)	*p* = .661
Parent income, *M* (*SD*)	6.83 (2.45)	7.37 (2.20)	6.90 (2.18)	6.43 (2.86)	6.96 (2.29)	*F* = 1.05, *p* = .370

a Chi-square only compared females and males since *n* < 10 for the group who picked “Other Identity.” ^b^Chi-square only compared those who picked White compared with those who picked another identity since *n* < 10 in most groups.

### Personality, Mental Health, and Substance Use

Table 2 shows average personality, mental health, and substance use scores for the total sample and by respondent category. Depression (1.12) and anxiety (1.34) scores suggest that, on average, participants were feeling anxious and depressed “less than 2 days” to “2-5 days” in the past 2 weeks. Personality scores fell slightly above the neutral midpoint of the scale for all traits (extraversion, neuroticism, agreeableness, conscientiousness, and openness). Impulsivity subscales showed average scores for negative and positive urgency between “disagree” and “neutral” while perseverance, premeditation, and sensation seeking scores were slightly higher, falling between “neutral” and “agree.” Participants reported using cannabis between “once a month” and “2-3 times per month” in the past year, on average. Average alcohol use in the past year fell between “once a week” and “2-6 times per week.” None of the tests of personality and mental health variables showed significant differences between responder types.

**Table 2.** Mental Health, Personality, Impulsivity, and Substance Use Measured at Baseline by Respondent Type.

	Total	Responder category				*F*-test
		Poor	Adequate	Good	Super	
	*M* (*SD*)	*M* (*SD*)	*M* (*SD*)	*M* (*SD*)	*M* (*SD*)	
Life satisfaction	3.07 (0.80)	3.14 (0.72)	3.20(0.81)	2.95 (0.84)	3.05 (0.78)	*F* = 1.07, *p* = .364
Depression	1.12 (0.55)	1.07 (0.50)	1.05 (0.55)	1.23 (0.56)	1.10 (0.55)	*F* = 1.16, *p* = .325
Anxiety	1.34 (0.76)	1.30 (0.61)	1.34 (0.75)	1.29 (0.76)	1.32 (0.85)	*F* = 0.05, *p* = .983
Personality						
Extraversion	3.18 (0.83)	3.24 (0.81)	3.34 (0.81)	3.08 (0.75)	3.10 (0.92)	*F* = 1.13, *p* = .336
Agreeableness	3.73 (0.70)	3.78 (0.72)	3.71 (0.78)	3.82 (0.65)	3.61 (0.67)	*F* = 0.91, *p* = .436
Conscientiousness	3.37 (0.59)	3.34 (0.67)	3.36 (0.58)	3.33 (0.60)	3.43 (0.54)	*F* = 0.29, *p* = .831
Neuroticism	3.17 (0.70)	3.07 (0.71)	3.16 (0.67)	3.14 (0.67)	3.25 (0.77)	*F* = 0.45, *p* = .718
Openness	3.69 (0.70)	3.59 (0.67)	3.73 (0.64)	3.58 (0.77)	3.82 (0.66)	*F* = 1.37, *p* = .254
Impulsivity						
Negative urgency	2.91 (0.93)	2.86 (0.94)	3.03 (0.94)	2.84 (0.95)	2.90 (0.89)	*F* = 0.43, *p* = .732
Perseverance	3.80 (0.71)	3.56 (0.90)	3.78 (0.65)	3.85 (0.65)	3.87 (0.70)	*F* = 1.45, *p* = .229
Premeditation	3.74 (0.67)	3.65 (0.60)	3.63 (0.61)	3.76 (0.73)	3.87 (0.66)	*F* = 1.36, *p* = .256
Sensation seeking	3.30 (0.90)	3.04 (0.95)	3.45 (0.89)	3.40 (0.90)	3.21 (0.86)	*F* = 1.75, *p* = .159
Positive urgency	2.39 (0.88)	2.24 (0.82)	2.62 (0.89)	2.37 (0.91)	2.27 (0.83)	*F* = 1.78, *p* = .152
Past-year cannabis	2.35 (1.56)	2.48 (1.48)	2.71 (1.79)	2.19 (1.58)	2.13 (1.31)	*F* = 1.58, *p* = .196
Past-year alcohol	3.23 (1.46)	3.34 (1.71)	3.43 (1.57)	3.02 (1.30)	3.23(1.40)	*F* = 0.80, *p* = .494

### Trends in Compliance

**Figure 1.**
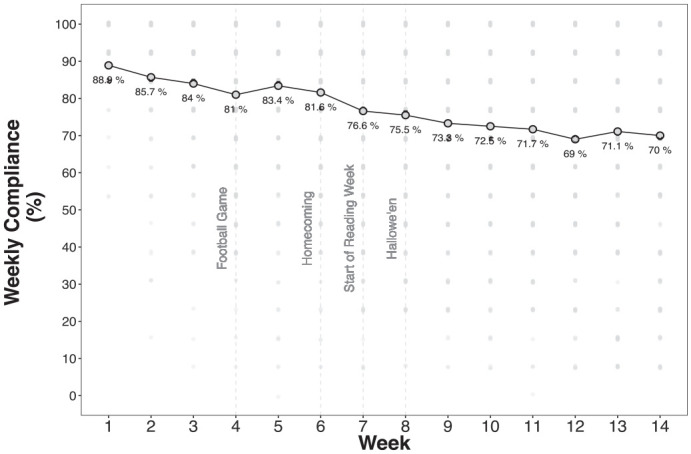
Weekly Study Compliance Rates.

**Figure 2.**
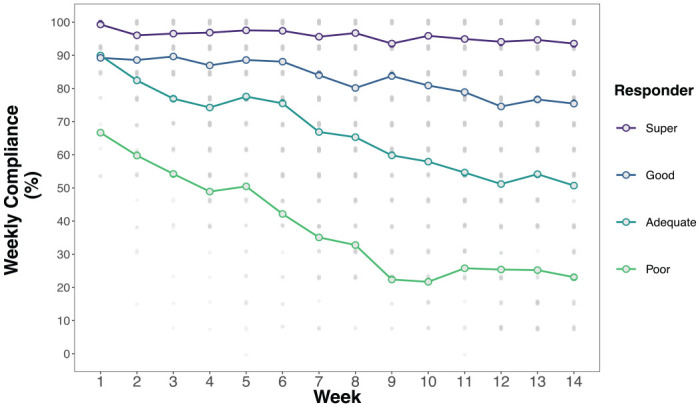
Weekly Compliance Rates by Responder Type.

**Figure 3.**
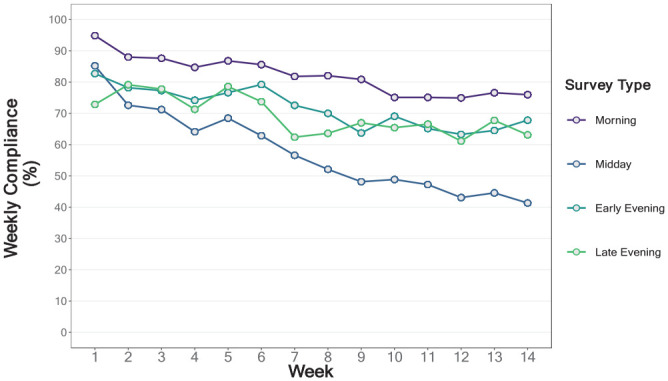
Weekly Compliance by Survey Type.

Figure 1 shows that average overall compliance gradually declined over the 14 weeks of data collection, from a high of 88.9% in Week 1 to 70% in Week 14. Mixed effects regression results predicting compliance rates showed significant main effects of Week (i.e., time), Responder Type, and Survey Type (complete model results in Supplement 2). As suggested by Figures 1, 2, and 3, two-way interactions between responder type, survey type, and survey week confirm that compliance rates and trends varied (a nonsignificant three-way interaction was removed to simplify the model). Simple slopes and contrast analyses are summarized in Tables 2, 3, and 4.

**Table 3.** Simple Slopes and Contrast Results Showing Rates of Change in Compliance by Responder Type, and Testing Differences in Rates of Change Between Responder Types.

	Estimate	*SE*	*z*	*p*
Compliance rate of change				
Poor	–3.13	.23	–13.79	<.001
Adequate	–1.83	.12	–15.27	<.001
Good	–0.81	.10	–7.90	<.001
Super	–.30	.10	–2.90	.015
Rate of change contrasts				
Poor-adequate	–1.30	.26	–5.06	<.001
Poor-good	–2.32	.25	–9.34	<.001
Poor-super	–2.83	.25	–11.37	<.001
Adequate-good	–1.03	.16	–6.52	<.001
Adequate-super	–1.54	.16	–9.73	<.001
Good-super	–0.51	.15	–3.52	.003

**Table 4.** Simple Slopes and Contrast Results for Average Wave Compliance Over 14 Weeks by Survey and Responder Type.

	Estimate	*SE*	*z*	*p*
Compliance rate of change				
Morning	–1.35	.12	–11.24	<.001
Midday	–2.28	.14	–16.61	<.001
Early evening	–1.27	.13	–10.18	<.001
Late evening	–1.17	.13	–9.41	<.001
Rate of change contrasts				
Morning-midday	0.93	.17	5.44	<.001
Morning-early	–0.07	.16	–0.45	1.00
Morning-late	–0.17	.16	–1.06	1.00
Midday-early	–1.00	.17	–5.81	<.001
Midday-late	–1.10	.17	–6.37	<.001
Early-late	–0.10	.17	–0.59	1.00

Table 3 shows that all responder categories had significant reductions in compliance over the 14 weeks, but reductions were steepest among less compliant participants (see Figure 2). For example, compliance rates among *poor* responders dropped by 3.13% per week on average, whereas *super* responders dropped by just 0.30% per week. Contrasts show that the rates of reduction over time are significantly different between each pair of responder groups.

Table 4 shows that all survey types had significant reductions in compliance over the 14 weeks, with no significant differences in reduction rates between the morning, early evening, and late evening surveys (on average, we observed a reduction of 1.17% to 1.35% per week for these surveys). In contrast, reductions over time in midday survey compliance were twice as large, 2.28% per week, and significantly larger than any other survey type (see Figure 3).

**Figure 4.**
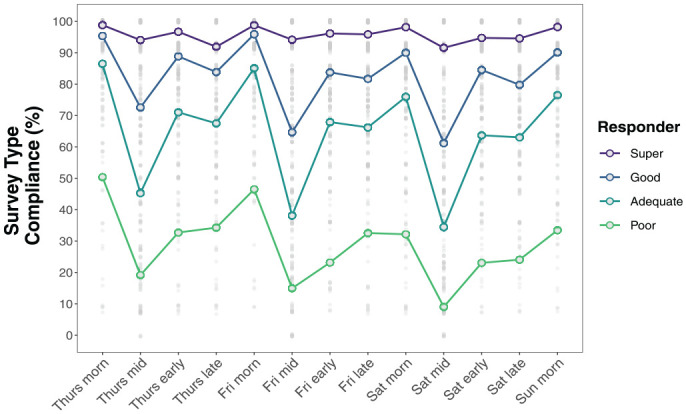
Within-Week Compliance Rates by Survey and Responder Type.

Finally, Table 5 shows that compliance rates were significantly higher for morning surveys compared with most other surveys for all responder groups. For example, poor responders completed 28% more morning surveys compared with midday and 7% more morning than early evening surveys. Among adequate and good responders, morning compliance was significantly higher than for all other survey types and midday compliance was significantly lower than for all other survey types. Early and late evening surveys had similar compliance rates in all responder groups. Super responders stood out as having fairly consistent compliance rates across surveys, showing just two statistically significant differences in compliance: Morning survey compliance was 5% higher than midday and 4% higher than late evening compliance in this group. Survey Type × Responder Compliance differences are illustrated in Figure 4.

**Table 5.** Simple Slopes and Contrast Results for Average Wave Compliance Over 14 Weeks by Survey and Responder Type.

	*M* (*SE*)_Poor_	*M* (*SE*)_Adequate_	*M* (*SE*)_Good_	*M* (*SE*)_Super_
Average weekly compliance				
Morning	60.9 (1.59)	83.2 (1.02)	93.3 (0.91)	98.5 (0.5)
Midday	32.0 (2.11)	55.4 (1.17)	74.7 (0.95)	93.4 (0.95)
Early evening	53.4 (1.82)	74.5 (1.05)	87.1 (0.91)	95.9 (0.95)
Late evening	57.5 (1.75)	73.5 (1.05)	86.2 (0.93)	94.1 (0.95)
	Est(*SE*)_Poor_	Est_Adequate_	Est_Good_	Est_Super_
Contrasts				
Morning-midday	28.85 (2.31)**	27.83 (1.29)**	18.67 (1.06)**	5.04 (1.07)**
Morning-early	7.41 (2.07)*	8.70 (1.18)**	6.24 (1.03)**	2.60 (1.07)
Morning-late	3.35 (2.01)	9.71 (1.18)**	7.16 (1.04)**	4.13 (1.07)*
Midday-early	–21.43 (2.45)**	–19.13 (1.31)**	–12.42 (1.06)**	–2.44 (1.07)
Midday-late	–25.49 (2.41)**	–18.12 (1.31)**	–11.51 (1.07)**	–0.91 (1.07)
Early-late	–4.06 (2.18)	1.01 (1.20)	–0.91 (1.04)	1.53 (1.07)

* *p* < .01. **p < .0001.

### Trends Over Time in Reminders Needed to Achieve Survey Completion

**Figure 5.**
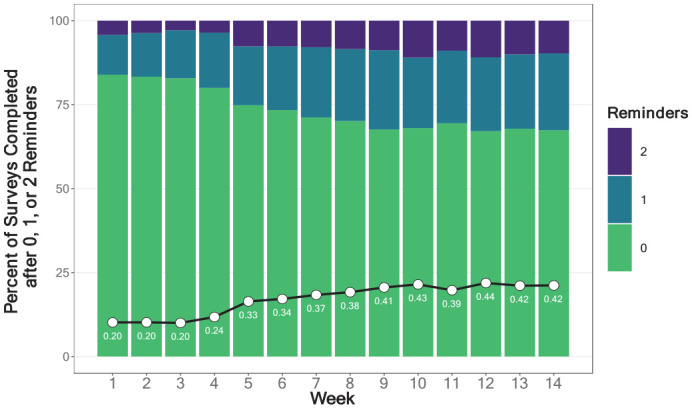
Need for Reminders: Percent of Completed Surveys Returned After 0, 1, or 2 Reminders by Week. *Note.* Line plot and text annotation shows increases in the mean number of reminders needed each week.

Figure 5 shows that among participants who completed surveys, the average number of reminders needed to achieve survey completion increased over time, from 0.20 in Week 1 (83.9% of surveys were submitted without a reminder) to 0.42 in Week 14 (67.4% of surveys were submitted without a reminder). Complete model results predicting reminders are available in Supplement 3 on our OSF project page (https://osf.io/d4ea8). We found significant effects of Week, Responder Type, Survey Type, and their two-way interactions (a nonsignificant three-way interaction was removed to simplify the model). Participants in all responder categories needed more reminders over time, and the need for reminders was higher in less-compliant versus more-compliant responder groups. There were no differences in need for reminders across survey types.

### Effects of Monetary Incentives

Table 6 shows summary statistics for rates of compliance on weeks preceding, during, and following a $25 daily draw win for the subset of *n* = 36 participants who won a daily draw. The random-effects ANCOVA (adjusting for study week) showed a significant effect of time, *F* = 5.30(2, 62.6), *p* = .007. Post hoc tests showed that compliance rates were lower on the weeks following a draw win, compared with weeks preceding, *t*(62.5) = 2.69, *p* = .009, and during, *t*(62) = 2.86, *p* = .006, a draw win. Compliance did not differ between weeks preceding and during a draw win, *t*(62.5) = .09, *p* = .93.

**Table 6.** Means and Standard Deviations for Participants Who Won a Daily Draw, Weeks 1 to 13.

	Pre-win	Win	Post-win
*M*_compliance_	76.65	80.34	73.72
*SD*_compliance_	18.90	20.76	25.50
*N*_compliance_	28	36	36

Table 7 shows summary statistics for rates of compliance on weeks before and after participants received a surprise bonus of $5, separated by responder type. A random-effects ANOVA of pre- to post-bonus compliance in Week 5 showed a significant reduction in compliance, *F* = 8.26(1,186), *p* = .005. Follow-up paired *t* tests separated by responder type isolated this effect to *adequate* responders, *t* = 2.64(186), *p* = .018. Compliance for the second $5 bonus was not significantly different pre-or post-bonus payment, *F* = 0.22(1,177), *p* = .64.

**Table 7.** Compliance Rates Before and After Unannounced Bonuses.

	Week 5 bonus		Week 10 bonus	
	Pre	Post	Pre	Post
Overall	**84.0%**	**81.1%**	76.0%	75.0%
By responder category				
Super	97.5%	97.1%	94.0%	96.0%
Good	89.1%	87.5%	83.1%	81.0%
Adequate	**78.2%**	**72.4%**	61.2%	57.2%
Poor	49.8%	43.8%	27.2%	28.7%

*Note.* Boldfaced values are significantly different pre- to post-bonus.

## Discussion

This study drew on data gathered from undergraduate alcohol drinkers to comprehensively examine variation in compliance with a long-term (14-weekend, 182-survey) ecological momentary assessment (EMA) protocol. Prior research focusing on study-level differences in compliance yielded few insights, leading us to focus on individual differences in the context of a single study. Three key findings emerged from our study. First, we found that compliance over time with our EMA protocol was not uniform. There was tremendous individual variability in rates of responding week-to-week, but variability was not explained by any sociodemographic, personality, mental health, or substance use measures collected at intake. Second, we found that compliance varied across our survey types and times of day, suggesting that different characteristics of our measurement contributed to better or worse compliance. Finally, we found no evidence that incentives enhanced or boosted compliance and draws may have *reduced* compliance.

### Compliance Over Time

Across all surveys administered, we achieved a compliance rate of 76.5%, declining from a high of 88.9% in the first week to 70% in the final week of surveys. Declines in compliance over time are consistent with other EMA and diary studies (Ono et al., 2019; Rintala et al., 2019; Silvia et al., 2013), but our mixed effects regression models show that rates of decline differ dramatically between people who are more versus less compliant, overall, with the EMA protocol. For example, *super* responders maintained a compliance rate above 90% over all 14 weeks, whereas *adequate* responders began at a compliance rate of 90% in Week 1 and declined to 51% in Week 14. Participants who completed 50% or more of their surveys over the course of the study started out with similar compliance rates but diverged quickly.

Reasons for these differences remain frustratingly elusive. We found no differences across responder categories on demographic measures (gender, ethnicity, educated parents, food insecurity), nor on any personality or well-being variables measured at study intake. Average *conscientiousness* scores, for example, varied by less than 0.1 points on a 5-point scale across responder types. These results are in line with other research that largely failed to detect any individual differences in compliance or missing reports (Murray et al., 2022; Sun et al., 2020). We also contradict Murray and colleagues, who identified that poorer self-regulation and higher substance use were weakly associated with poorer compliance. In a supplemental analysis inspired by an anonymous reviewer’s comment, we compared our participants with the 43 people who completed our intake survey, passed our screening measures, but decided not to enroll in the EMA study phase. Findings are summarized in Supplement 4 (available on our OSF project page) and show that samples did not differ on 15 of 21 demographic, personality, or well-being measures tested (exceptions were extraversion, neuroticism, negative urgency, past year cannabis use, life satisfaction, and gender). Considering the damaging role that bias can play in EMA data quality (van Berkel et al., 2020), these findings are reassuring. At least among people who agree to enroll in a demanding EMA study, those who contribute less data do not appear to differ from their counterparts who contribute more data on important traits and demographic characteristics. Our supplemental analysis suggests that some characteristics may contribute to the decision to enroll or not in an EMA study, but this question requires further study beyond the scope of our exploratory work. Once enrolled, reasons for compliance may lie in participants’ experiences and engagement with the study protocol itself.

We took several steps to ensure that participants enrolled into our EMA study phase were interested and engaged at the outset of the study. Everyone who enrolled had to first decide to complete our eligibility screen, provide contact information, respond to an invitation to complete our intake survey, express interest in continuing to participate, complete a secondary consent form, provide their phone number, and respond to a confirmation text message. We assume that participants willing to complete these numerous steps anticipated a favorable cost-benefit trade-off to participating and found our team to be trustworthy (see Dillman et al., 2014, Chapter 2). Thereafter, participants likely had mixed reactions to the surveys and the protocol itself. In follow-up interviews to their EMA protocol, Eisele and colleagues (2022) found that some participants described the protocol becoming burdensome as initial excitement wore off and questions became boring. Others reported getting used to survey prompts as part of their daily routine. Participants in one study who had lower overall compliance were more likely to respond to morning surveys, if they were *not* already using their phone when a prompt was received, and if they tended to use a lot of apps on their phone (van Berkel et al., 2020). In contrast, participants in that study with high compliance rates responded to prompts irrespective of context. In the present study, this appeared to be the case for *super* respondents, who comprised 29% of our sample. They missed very few prompts and declined very little in compliance over time. Their need for reminders each week was also lower than other groups and increased at a slower rate. Habituation to the protocol and waning novelty might explain compliance reductions over time among our poor, adequate, and good responders. Text message alerts may also have become easier to ignore, explaining the increased need for reminders. Identifying strategies that can increase the size of a *super*-like group in EMA studies is a high priority for limiting missing data, and the characteristics and timing of individual surveys may be useful points of intervention.

### Survey Type Differences

A key finding was that participants were selective in their survey completion, with some surveys apparently easier or more desirable to complete. Consistent with other research (van Berkel et al., 2020), *morning* surveys had the highest completion rates in all groups, including the poor responders. These surveys contained more questions than any other survey but were available for 4 hours and during the earlier part of the day when respondents are less occupied with social and household obligations, presumably leaving fewer barriers to participation. Indeed, we selected the 10:00 a.m. to 2:00 p.m. timeframe with undergraduates’ typical daily schedules in mind. EMA studies of community samples and older adults, for example, might achieve higher compliance with survey prompts released at 6:00 or 7:00 a.m.. Early and late evening surveys were completed at the second-highest frequency, and at virtually identical rates. Despite the 2-hour window for the early evening survey versus 4-hour window for the late evening survey (which began at 11:00 p.m.), compliance rates for these surveys were not significantly different in any responder group. Midday surveys stood out as having the poorest compliance rates, with dramatic differences from all other survey types in every group except *super* respondents. For good, adequate, and poor respondents, compliance rates dropped by 20 to 40 percentage points on average between the morning and midday surveys on the same day (see Figure 5).

The midday survey was available for just 2 hours. This was an adequate completion window for the early evening survey, but its presentation during the transitional part of the day when many people are commuting home and preparing for evening social activities might have been a disadvantage. Perhaps more importantly, however, the midday survey involved a tedious cognitive task with a median completion time of just over 4 minutes. We suspect that large numbers of participants regularly chose to skip this survey, on which they were asked to complete a response inhibition (Stop Signal) task in 5 blocks, with 16 trials per block. Our initial expectation was that participants would treat this task as a game, which included accuracy feedback after each block. In other research, a word-ranking task with “gamified” elements (e.g., score, player leaderboard, time challenge) achieved a participation rate nearly double that of a nongamified version of the same task (van Berkel et al., 2017). Unfortunately, our midday task was likely more burdensome than fun, leading to reduced compliance when combined with the potential challenge of wresting participants’ attention at a busy time of day.

### Incentives May Delay Attrition

At the outset of this study, we anticipated that regularly delivered incentives would help to overcome feelings of burden associated with continued EMA participation and at times that surveys felt uninteresting or intrusive. Our design included $1 compensation per survey, with gift card payments issued by email 2 days after each weekend survey burst (most participants redeemed or uploaded their payments to Amazon immediately). Regular incentives are a feature that likely contributed to willingness to participate and self-selection into our study (Ludwigs et al., 2020), but our unannounced bonuses and gift card draws allowed us to directly test the impact of incentives on compliance. For both types of incentive, we found no evidence that compliance was “boosted” the week following a bonus or a draw win. On the contrary, compliance rates were significantly lower on weeks after students won a gift card draw.

Extrapolating from principles of economic exchange, we assume that people will be more willing to complete work (responding to surveys) if they are compensated for that work (cash or gift cards). Meta-analytic work bears this out, showing that compliance rates are higher in studies offering financial incentives (e.g., Church, 1993; Wrzus & Neubauer, 2022), and people who feel adequately compensated tend to complete more surveys (Martinez et al., 2021). In contrast, unannounced bonuses and prize winnings ought to evoke participants’ sense of social exchange obligation (Dillman et al., 2014, p. 370) to boost compliance. In paper-based mail surveys, including a small “pre-incentive” with the initial survey invitation increased participation rates by 11% to 25% compared with control conditions in which invitations included no pre-incentive (Lesser et al., 2001). We found no evidence that participants completed more surveys in the weeks following receipt of a surprise $5 bonus. However, compliance rates also did not *decline* the week after bonuses were received, leaving open the possibility that these incentives delayed compliance reductions. Our subset of draw-winning participants actually completed fewer surveys after winning a $25 gift card, suggesting that draw winnings might have supplanted motivation to complete the following week’s surveys. However, the overall compliance rate for participants who won a draw (77.2%) was not significantly different from the overall compliance rate for participants who did not win a draw (74.5%), *t*(65.51) = –.82, *p* = .415, suggesting no persistent effect of winning draws on engagement. Incentive effectiveness in EMA studies is a topic needing more careful study, but we agree with Kaurin and colleagues (2022) who advised the important first step of reporting study design decisions in detail, including incentive structures.

### Strengths and Limitations

A key strength of the present study was our use of detailed information about compliance in a longitudinal EMA design that permitted a contrasting perspective to the almost exclusively between-study effects that have been tested to date. We were able to evaluate compliance trends over a 14-week, 182-survey period; test for sociodemographic, personality, mental health, and substance use differences in compliance over time; identify differences across survey types and times of day; and evaluate different incentive techniques to maintain compliance. Our overall compliance rate of 76.5% across 14 weeks is comparable with what numerous other studies managed with just 1 or 2 weeks of data collection. In other words, we were able to leverage a strong sample with diverse design features to test exploratory questions about within-study variation in EMA protocol compliance.

At the same time, compliance data were a by-product of this sample, not the primary goal. We did not randomly assign participants to receive different kinds of surveys or different incentive structures, and so are limited in some of the causal conclusions we can draw from this work. An unanticipated limitation was participants’ apparent dislike for the Stop-Signal task that figured heavily on the midday survey. We presume, rather than confirm, that low compliance on this survey was due to the nature of the task, but we cannot rule out that time of day alone (4:00 p.m.–6:00 p.m.) may have driven low compliance.

### Conclusion and Recommendations

In sum, this 14-week, 182-survey EMA protocol showed gradually waning compliance over time that differed by type of respondent and type of survey. Demographics, personality, mental health, and substance use measures did not distinguish between participants with higher and lower rates of compliance across the study. From these findings, we offer the following recommendations for future EMA research:

#### Develop Study Enrolment Strategies to Maximize the Share of Highly Engaged Participants

Our *super* respondents completed nearly every survey they were given. Their rate of reduction in compliance over time was also slower than every other group, suggesting that highly engaged participants can maintain good response rates even in long protocols. Given the critical importance of high-quality complete data in EMA designs, research teams need to make every effort to ensure that the people who end up in the sample *want* to be in the study. It can be challenging to entice prospective participants to enroll in a study with numerous follow-ups, so our first recommendation appears counterintuitive: make it at least a little bit difficult to sign up for the study. Only enroll participants who complete multiple screenings, check-ins, or other stages of recruitment prior to beginning an EMA survey sequence. Prospective participants dropped off at every stage of our screening procedure, leaving us with an eventual sample that produced a high compliance rate over many weeks. This strategy has the added benefit of creating opportunities to detect and discard “bots” and careless respondents that have in recent years become the scourge of online data collection (Storozuk et al., 2020). Focusing on genuine participants, our subsequent recommendations include study design suggestions that might help to hold their engagement levels throughout a study.

#### Administer Short Surveys and Tasks That Are Fun (or at Least not Tedious) to Complete

We dispute the conclusion from other work that survey length or depth does not affect protocol compliance. Although meta-analyses and experimental data preceding our work failed to find any between-study differences in compliance related to surveys with more questions (Hasselhorn et al., 2022; Ottenstein & Werner, 2022; Williams et al., 2021), evidence suggests that perceived burden is higher and data quality may be poorer (Eisele et al., 2022; Hasselhorn et al., 2022). Our midday survey cognitive task was distinct from our other surveys and achieved much poorer compliance rates for all but the most engaged *super* respondents. We recommend that research teams pay careful attention to features of a survey or task that might increase perceived burden on repetition (lengthy surveys, boring tasks, seeing the same questions over and over). Strategies such as random item selection from a pool of questions and including interactive games with feedback may help to break up some of the tedium of frequent responding.

#### Pay Participants and Consider Low-Cost Supplemental Incentives to Delay Attrition

Incentives are an important tool for engaging participants in an EMA protocol. The high overall compliance rate in our study is likely attributable in part to its incentive structure and frequent payment schedule. We did not see a boost to compliance rates in weeks following our supplemental surprise bonuses, but we nonetheless recommend this approach as a potential strategy to forestall some attrition. Over the course of the study, we spent over $25,000 on weekly incentives and just $3,000 in bonuses and draws. Given their potential to build goodwill, enhance trust, and maintain participant engagement (if not enhance it), the cost-benefit trade-off still favors the use of supplemental incentives, although we are cautious about the use of higher-value, lottery-like prizes that showed short-term compliance reductions in this study. We encourage research teams to think of creative ways to incorporate supplements into their own study designs.

Participant engagement in EMA research is undoubtedly difficult to sustain over time, and every new survey brings an opportunity for more people to drop out or ignore prompts. Kaurin and colleagues (2022) offer recommendations for balancing the density, depth, and duration of EMA assessments. We further encourage research teams to carefully build into their study design opportunities to enhance engagement. Reminder messages, feedback after completing surveys, supplemental incentives, and gamified elements are promising tools to enhance data quantity and quality.

## Notes

1. This rate was obtained by searching the Scopus database for instances of “ecological momentary assessment” or “experience sampling” or “ambulatory assessment” and, for each year, taking the ratio of the number of articles including these keywords to the total number of articles in the database. In 2008: Keywords in 0.00542% of all articles. In 2021: Keywords in 0.0342% of all articles. 0.0342/0.00542 = 6.31.

2. Students estimated their parents’ incomes within ranges (e.g., 6 = $80,000–$100,000; 7 = 100,001–$125,000). For a sample mean income of 6.83 (in between two categories), we multiplied the maximum from the lower category ($100,000 for category “6”) by the difference between the higher category code and the sample mean (7 – 6.83 or 0.17) and added the maximum from the higher category ($125,000 from category “7”) multiplied by the difference between the sample mean and the lower category code (6.83 − 6 or 0.83). Summing these two values returns a “weighted maximum” estimated income: ($100,000 × 0.17 = $17,000) + ($125,000 × 0.83 = 103,750) = $120,750.

## References

[bibr1-10731911231159937] Andresen E. M. Malmgren J. A. Carter W. B. Patrick D. L. (1994). Screening for depression in well older adults: Evaluation of a short form of the CES-D. American Journal of Preventive Medicine, 10(2), 77–84. 8037935

[bibr2-10731911231159937] Bates D. Mächler M. Bolker B. Walker S. (2015). Fitting linear mixed-effects models using lme4. Journal of Statistical Software, 67(1), 1–48. 10.18637/jss.v067.i01

[bibr3-10731911231159937] Bedard C. King-Dowling S. McDonald M. Dunton G. Cairney J. Kwan M. (2017). Understanding environmental and contextual influences of physical activity during first-year University: The feasibility of using ecological momentary assessment in the MovingU study. JMIR Public Health and Surveillance, 3(2), e7010. 10.2196/publichealth.7010 PMC5471502 28566264

[bibr4-10731911231159937] Burke L. E. Shiffman S. Music E. Styn M. A. Kriska A. Smailagic A. Siewiorek D. Ewing L. J. Chasens E. French B. Mancino J. Mendez D. Strollo P. Rathbun S. L. (2017). Ecological momentary assessment in behavioral research: Addressing technological and human participant challenges. Journal of Medical Internet Research, 19(3), e77. 10.2196/jmir.7138 PMC5371716 28298264

[bibr5-10731911231159937] Church A. H. (1993). Estimating the effect of incentives on mail survey response rates: A meta-analysis. Public Opinion Quarterly, 57, 62–79.

[bibr6-10731911231159937] Csikszentmihalyi M. Larson R. (1987). Validity and reliability of the experience-sampling method. Journal of Nervous & Mental Disease, 175, 526–537. 3655778 10.1097/00005053-198709000-00004

[bibr7-10731911231159937] Diener E. Emmons R. A. Larsen R. J. Griffin S. (1985). The satisfaction with life scale. Journal of Personality Assessment, 49, 71–75. 16367493 10.1207/s15327752jpa4901_13

[bibr8-10731911231159937] Dillman D. A. Smyth J. D. Christian L. M. (2014). Internet, phone, mail, and mixed-mode surveys: The tailored design method. John Wiley & Sons.

[bibr9-10731911231159937] Eisele G. Vachon H. Lafit G. Kuppens P. Houben M. Myin-Germeys I. Viechtbauer W. (2022). The effects of sampling frequency and questionnaire length on perceived burden, compliance, and careless responding in experience sampling data in a student population. Assessment, 29(2), 136–151. 32909448 10.1177/1073191120957102

[bibr10-10731911231159937] Harari G. M. Müller S. R. Mishra V. Wang R. Campbell A. T. Rentfrow P. J. Gosling S. D. (2017). An evaluation of students’ interest in and compliance with self-tracking methods. Social Psychological and Personality Science, 8(5), 479–492.

[bibr11-10731911231159937] Hasselhorn K. Ottenstein C. Lischetzke T. (2022). The effects of assessment intensity on participant burden, compliance, within-person variance, and within-person relationships in ambulatory assessment. Behavior Research Methods, 54(4), 1541–1558. 34505997 10.3758/s13428-021-01683-6 PMC9374628

[bibr12-10731911231159937] Jones A. Remmerswaal D. Verveer I. Robinson E. Franken I. Wen C. Field M. (2019). Compliance with ecological momentary assessment protocols in substance users: A meta-analysis. Addiction, 114(4), 609–619. 10.1111/add.14503 30461120 PMC6492133

[bibr13-10731911231159937] Kaurin A. King K. Wright A. G. (2022). Studying personality pathology with ecological momentary assessment: Harmonizing theory and method. https://psyarxiv.com/chszq/10.1037/per0000596 36848074

[bibr14-10731911231159937] Kuznetsova A. Brockhoff P. B. Christensen R. H. B. (2017). LmerTest package: Tests in linear mixed effects models. Journal of Statistical Software, 82(13), 1–26. 10.18637/jss.v082.i13

[bibr15-10731911231159937] Lenth R. V. (2022). emmeans: Estimated marginal means, aka least-squares means (R package version 1.7.4-1). https://CRAN.R-project.org/package=emmeans

[bibr16-10731911231159937] Lesser V. M. Dillman D. A. Carlson J. Lorenz F. Mason R. Willits F. (2001). Quantifying the influence of incentives on mail survey response rates and their effects on nonresponse error [Paper presentation]. Proceedings of the Annual Meeting of the American Statistical Association, Atlanta, GA, United States.

[bibr17-10731911231159937] Ludwigs K. Lucas R. Veenhoven R. Richter D. Arends L. (2020). Can happiness apps generate nationally representative datasets? A case study collecting data on people’s happiness using the German Socio-economic Panel. Applied Research in Quality of Life, 15, 1135–1149. 10.1007/s11482-019-09723-2

[bibr18-10731911231159937] Martinez G. J. Mattingly S. M. Robles-Granda P. Saha K. Sirigiri A. Young J. Chawla N. De Choudhury M. D’Mello S. Mark G. Striegel A. (2021). Predicting participant compliance with fitness tracker wearing and ecological momentary assessment protocols in information workers: Observational study. JMIR Mhealth and Uhealth, 9(11), e22218. 34766911 10.2196/22218 PMC8663716

[bibr19-10731911231159937] Munger A . (2016). 2016 census highlights: Factsheet 7. https://www.fin.gov.on.ca/en/economy/demographics/census/cenhi16-7.html

[bibr20-10731911231159937] Murray A. L. Yang Y. Zhu X. Speyer L. G. Brown R. Eisner M. Ribeaud D. (2022). Respondent characteristics associated with compliance in a general population ecological momentary assessment study. Advance online publication. 10.31234/osf.io/jadwq PMC10698810 37184112

[bibr21-10731911231159937] Ono M. Schneider S. Junghaenel D. U. Stone A. A. (2019). What affects the completion of ecological momentary assessments in chronic pain research? An individual patient data meta-analysis. Journal of Medical Internet Research, 21(2), e11398. 30720437 10.2196/11398 PMC6379815

[bibr22-10731911231159937] Ottenstein C. Werner L. (2022). Compliance in ambulatory assessment studies: Investigating study and sample characteristics as predictors. Assessment, 29(8), 1765–1776. 34282659 10.1177/10731911211032718 PMC9597150

[bibr23-10731911231159937] R Core Team. (2021). R: A language and environment for statistical computing. R Foundation for Statistical Computing. https://www.R-project.org/

[bibr24-10731911231159937] Rintala A. Wampers M. Myin-Germeys I. Viechtbauer W. (2019). Response compliance and predictors thereof in studies using the experience sampling method. Psychological Assessment, 31(2), 226–235. 30394762 10.1037/pas0000662

[bibr25-10731911231159937] Selker R. Love J. Dropmann D. Moreno V. (2021). jmv: The “jamovi” analyses (R package version 2.0). https://CRAN.R-project.org/package=jmv

[bibr26-10731911231159937] Shiffman S. Stone A. A. Hufford M. R. (2008). Ecological momentary assessment. Annual Review of Clinical Psychology, 4, 1–32. 10.1146/annurev.clinpsy.3.022806.091415 18509902

[bibr27-10731911231159937] Silvia P. J. Kwapil T. R. Eddington K. M. Brown L. H. (2013). Missed beeps and missing data: Dispositional and situational predictors of nonresponse in experience sampling research. Social Science Computer Review, 31(4), 471–481.

[bibr28-10731911231159937] Soto C. J. John O. P. (2017). Short and extra-short forms of the Big Five Inventory–2: The BFI-2-S and BFI-2-XS. Journal of Research in Personality, 68, 69–81.

[bibr29-10731911231159937] Spitzer R. L. Kroenke K. Williams J. B. Löwe B. (2006). A brief measure for assessing generalized anxiety disorder: The GAD-7. Archives of Internal Medicine, 166, 1092–1097. 16717171 10.1001/archinte.166.10.1092

[bibr30-10731911231159937] Storozuk A. Ashley M. Delage V. Maloney E. A. (2020). Got bots? Practical recommendations to protect online survey data from bot attacks. The Quantitative Methods for Psychology, 16(5), 472–481.

[bibr31-10731911231159937] Sun J. Rhemtulla M. Vazire S. (2020). Eavesdropping on missing data: What are university students doing when they miss experience sampling reports? Personality and Social Psychology Bulletin, 47(11), 1535–1549. 33342369 10.1177/0146167220964639

[bibr32-10731911231159937] van Berkel N. Goncalves J. Hosio S. Kostakos V . (2017). Gamification of mobile experience sampling improves data quality and quantity. Proceedings of the ACM on Interactive, Mobile, Wearable, and Ubiquitous Technologies, 1(3), 1–21.

[bibr33-10731911231159937] van Berkel N. Goncalves J. Hosio S. Sarsenbayeva Z. Velloso E. Kostakos V . (2020). Overcoming compliance bias in self-report studies: A cross-study analysis. International Journal of Human-Computer Studies, 134, 1–12.

[bibr34-10731911231159937] Whiteside S. P. Lynam D. R. (2001). The five-factor model and impulsivity: Using a structural model of personality to understand impulsivity. Personality and Individual Differences, 30, 669–689.

[bibr35-10731911231159937] Wickham H. (2016). ggplot2: Elegant graphics for data analysis. Springer-Verlag.

[bibr36-10731911231159937] Wickham H. François R. Henry L. Müller K. (2022). dplyr: A grammar of data manipulation. https://dplyr.tidyverse.org, https://github.com/tidyverse/dplyr

[bibr37-10731911231159937] Williams M. T. Lewthwaite H. Fraysse F. Gajewska A. Ignatavicius J. Ferrar K. (2021). Compliance with mobile ecological momentary assessment of self-reported health-related behaviors and psychological constructs in adults: Systematic review and meta-analysis. Journal of Medical Internet Research, 23(3), e17023. 10.2196/17023 PMC7970161 33656451

[bibr38-10731911231159937] Wrzus C. Neubauer A. B. (2022). Ecological momentary assessment: A meta-analysis on designs, samples, and compliance across research fields. Assessment. Advance online publication. 10.1177/10731911211067538 PMC9999286 35016567

